# The future of radiology is now: the first 100 articles published in *European Radiology Experimental*

**DOI:** 10.1186/s41747-019-0106-5

**Published:** 2019-07-26

**Authors:** Francesco Sardanelli

**Affiliations:** 1 Department of Biomedical Sciences for Health, Università degli Studi di Milano, Milan, Italy; 2 Unit of Radiology, IRCCS Policlinico San Donato, Via Morandi 30, 20097 San Donato Milanese, Milan, Italy

**Keywords:** Artificial intelligence, European Radiology Experimental, Models (animal), Phantoms (imaging), Research design

## Abstract

*European Radiology Experimental* reached the first 100 articles published in two years. Rejection rate was 30%, publication rate increased from 3.5/month in the first 12-month period to 4.8/month in the second 12-month period. The journal metrics were: 25 days from submission to first decision, 96 days from submission to acceptance, and 69 days from acceptance to publication. At the end of May 2019, we accumulated a total of 82,367 article accesses, 541 Altmetric score, and 110 citations for 92 published articles. Europe accounted for 85% of article origin. One third of corresponding authors were not radiologists/radiology residents, but were rather mainly physicists, engineers, or computer scientists. The distribution among subspecialties/body parts was well balanced; 9% of the topics regarded patient’s safety, radioprotection, or contrast media. Magnetic resonance imaging (MRI) and computed tomography (CT) accounted for 71% of the articles. Twenty-two percent of original articles/technical notes reported on animal models, 15% on phantoms, 3% on *in silico*, 2% on human cadavers, and 2% on cells. Nine articles regarded artificial intelligence and/or radiomics, and 2 regarded augmented reality. Of 100 articles, 57 declared funding sources. A total of 517 independent reviews were performed by 92 reviewers. The five articles quoted the most regarded augmented reality, spectral photon-counting CT, artificial intelligence, MRI radiomics, and diffusion tensor imaging of the musculoskeletal and peripheral nerve systems. The journal is complying with aims and scope of its “experimental” profile.

## Introduction

In 2016, the European Society of Radiology (ESR) decided to launch *European Radiology Experimental*, a new journal belonging to the ESR journal family. It had to show a different profile than that of *European Radiology*, which is mainly devoted to presenting results of radiological research to be applied in clinical practice, and of *Insights into Imaging*, which is mainly devoted to presenting educational articles, statements, position papers, and critical reviews. The adjective “experimental” had to mark the profile of the journal. The first articles were published on June 29, 2017.

In the “Aims and scope” declared in the website [1], we outlined the journal mission: “to adhere to the multidisciplinary paradigm of the 21^st^ century, fostering a strong connection between radiology in the experimental setting and basic science”. We expected research articles on phantoms, animal models, new imaging modalities and techniques, novel contrast materials, tracers, and probes, three-dimensional modelling/printing, novel image reconstruction algorithms and post-processing, computer-assisted detection/diagnosis, imaging biomarkers, radiomics/radiogenomics, artificial intelligence, and innovations for interventional radiology. The goal of the journal was “to provide a forum open not only to radiologists, nuclear physicians, or radiation therapists but also to other professionals such as physicists, biologists, chemists, bioengineers, biomathematicians, experts in computer science, information technology, and bioinformatics, as well as physicians working in medical imaging from other medical specialties such as pathologists, geneticists, neurologists, surgeons, cardiologists, and many more”. We tried to fit with these aims by composing an Editorial Board (EB) that includes non-radiologists who currently represent more than one third (36%) of the members.

Following the trend of scientific publishing towards a higher accessibility of the results of research [2], similarly to *Insights into Imaging*, *European Radiology Experimental* was created as an online only, open access journal, with the big advantage of having ESR coverage of the article processing charge available for active ESR members that do not belong to institutions already providing the coverage because of a special agreement with the publisher.

In approximately two years, the journal reached the milestone of the first 100 articles published. In the meantime, it has been officially indexed by: Elton B. Stephens Co. Discovery Service [3]; Directory of Open Access Journals [4]; Google Scholar [5]; International Nuclear Information System Atomindex [6]; Medline [7]; Online Computer Library Center WorldCat Discovery Service [8]; ProQuest, ExLibris Primo [9]; ProQuest, ExLibris Summon [10]; PubMedCentral [11].

In this Editorial, these 100 articles are evaluated in order to compare the expectations with the real trend of the journal. As we will see, these 100 articles offer a view on ongoing innovations in radiology.

## Submission, publication, rejection rate, and journal metrics

The first 100 articles were published from the end of June 2017 to approximately the same date in 2019. Overall, in this time period, we had 142 submissions and 42 rejections resulting in a rejection rate of 30%. The rate of publication raised from about 3.5/month in the first 12-month period to about 4.8/month in the second one. In 2018, the only year we can entirely evaluate, this rate was 3.8, and in the first half of 2019, it was 4.5. The trend is positive.

The journal metrics of manuscript processing are the following (average data): 25 days to first decision for all manuscripts (30 days for reviewed manuscripts only); 96 days from submission to acceptance (including editorial editing); 69 days from acceptance to publication. Thus, for the interval time mainly depending on the reviewing/editorial side (time to first decision and time from acceptance to publication), we have on average about three months of waiting time. These times may be reduced but compare favourably with other journals in the field of medical imaging.

At the end of May 2019, for 92 published articles, we accumulated a total of 82,367 article accesses, 541 Altmetric^1^ score, and 110 citations. Thirty-eight articles were quoted from 1 to 17 times. The trend of new citations was about 4/month during 2018, and about 13/month during the first 5 months of 2019. This citation trend is a good promise for the future.

## Geographic and professional origin of articles

Table 1 shows the geographic origin of the corresponding authors. As expected, Europe was the major contributor, with Italy, Germany, Switzerland, and The Netherlands each providing over 10 articles, ranking them at the top as well. Interestingly, North America and Asia each provided 7 articles, creating a balance between them.

**Table 1:** The first 100 articles published in *European Radiology Experimental*: geographic origin of the corresponding authors

Country	Number
Italy	22
Germany	14
Switzerland	12
The Netherlands	11
France	8
United Kingdom	6
United States	6
Ireland	5
China	4
Spain	3
Japan	2
Others^a^	7
Total	100
Geographic area	Number
Europe	85
North America	7
Asia	7
Africa	1
Total	100

^a^One article per each of the following: Austria, Belgium, Canada, Egypt, Finland, South Korea, Sweden

Table 2 shows the diversity in professional profiles of the corresponding authors. More than half of them were radiologists, and 15 were radiology residents (showing that the journal is attractive for the next generation of radiologists). In agreement with the aims and scope of the journal, the remaining 32 authors were other professionals, notably physicists, engineers, and computer scientists. Overall, about one third of corresponding authors were neither radiologists nor radioology residents.

**Table 2:** The first 100 articles published *in European Radiology Experimental*: professional profile of the corresponding authors

Professional profile	Number
Radiologist	53
Radiology resident	15
Physicist	10
Engineer/Computer scientist	8
Radiology technician	3
Other professional^a^	11
Total	100

^a^Neuroscientist, biologist, chemist, nuclear physician, other medical doctor, etc

## Article type, content, and funding

Important issues to be analysed are the type and content of the articles as well as the funding, if applicable, which supported the research.

Article types, the involvement of living humans *versus* more “experimental” models, and the imaging techniques utilised are reported in Tables 3, 4, and 5, respectively. As expected, original articles (*n* = 73) and technical notes (*n* = 15) revealed new data or preliminary results of new approaches. The distribution among subspecialties/body parts was well balanced, ranging from 7% of Thorax/Lung to 14% of Abdomen/Pelvis and Neuro/Head-neck/Spine, indicating that we are attracting articles from the whole world of medical imaging. Of note, 9% of the articles regarded topics dealing with patient’s safety, radioprotection, or contrast media, showing that experimental research is ongoing also on the patient-centred perspective. Magnetic resonance imaging (MRI) and computed tomography (CT) accounted for 71% of the articles.

**Table 3:** The first 100 articles published in *European Radiology Experimental*: article types

Article type	Number
Original article	73
Technical note	15
Narrative review	6
Editorial	2
Methodology	2
Systematic review	1
Hypothesis	1
Total	100

**Table 4:** The first 100 articles published in *European Radiology Experimental*: involved subspecialties/body parts

Subspecialty/Body parts	Number	Percentage
Abdomen/Pelvis	16	14%
Neuro/Head-neck/Spine	16	14%
Interventional	14	12%
Cardiovascular	12	10%
Musculoskeletal	11	9%
Oncology (general)	11	9%
Safety/Radioprotection/Contrast media	10	9%
Breast	9	8%
Thorax/Lung	8	7%
General/Other	9	8%
Total	116	100%

The total is over 100, due to the possibility of multiple subspecialties/body parts involved in one article

**Table 5:** The first 100 articles published in *European Radiology Experimental*: most involved imaging techniques

Imaging technique	Number	Percentage
Magnetic resonance	46	41%
Computed tomography	33	30%
X-ray/Mammography	9	8%
DSA/Fluoroscopy	11	10%
Ultrasound	6	5%
Optical imaging	2	2%
Positron emission tomography	2	2%
Dual x-ray absorptiometry	1	1%
Single-photon emission tomography	1	1%
Total	106	100%

The total is over 100, due to the possibility of multiple techniques involved in one article

Table 6 specifically testifies we are fitting with our “experimental” mission. Forty-four percent of original articles and technical notes were not performed on living humans: 22% on animal models, 15% on phantoms, 3% on *in silico*, 2% on human cadavers, and 2% on cells. As a special note, we highlight that 9 articles regarded artificial intelligence and/or radiomics, while 2 regarded augmented reality.

**Table 6:** The first 100 articles published in *European Radiology Experimental*: studies on living humans and studies on other models

Study object of original articles and technical notes	Number	Percentage
Living humans	49	56%
Human cadavers	2	2%
Cells	2	2%
Animal models	19	22%
Phantoms	13	15%
*In silico*	3	3%
Total	88	100%

Another interesting aspect is the declared funding, shown in Table 7. It is worth noting that 57 articles had funding sources, indicating that the published data came from institutions which play a substantial role in the international medical imaging research network. The most frequent funding sources were: European Commission and European Research Council, European Union; German Research Foundation and Federal Ministry of Education and Research, Germany; National Heart Lung and Blood Institute, United States; National Institute for Health Research and Medical Research Council, United Kingdom; Ministry of Health, Italy.

**Table 7:** The first 100 articles published in *European Radiology Experimental*: funding declarations

Funding	Number
Public	42
Private	8
Public and private	7
None declared	43
Total	100

## The peer-review process

The peer-review process is the main core of a journal. The quality of the reviewers is the key factor of success. Multiple models are available today for the review process: single- or double-blind; open; collaborative; post-publication, etc. In my opinion, in a field as large as medical imaging (even though the tendency toward subspecialisation and hyper-specialisation can create relatively narrow research areas), the double-blind review process remains the best option, limiting biases that are implicitly associated with any judgement of a manuscript. For a medical journal, the condition of good results is not only a good-high quality of reviewers, but also a short review time. As mentioned above, we managed to keep this time acceptable.

This was only possible thanks to a tremendous work performed by the reviewers, both members and non-members of the EB. I thanked them during the Editorial Board meeting at the European Congress of Radiology 2019 in Vienna. However, I always think that their work is too often obscured and never sufficiently acknowledged. To have 100 articles published, we had 517 independent reviews (423 for the accepted articles, 94 for the rejected articles) from 92 different reviewers. I want to express my gratitude to all of them here, in particular to those who gave their working time to the journal without being EB members, and contributed with ten or more reviews. These colleagues deserve a warm word of thanks: *Giovanni Mauri*, Milan; *Francesco Secchi*, Milan; *Stefania Rizzo*, Lugano; *Martin Beeres*, Frankfurt; *Annamaria Ierardi*, Milan; *Fabio Galbusera*, Milan; and *Julian Wichmann*, Frankfurt.

## The five most quoted articles: the future is now

Using the data available from the journal website at the end of May 2019 for the first 92 articles, we obtained high Pearson linear correlation coefficients for the number of citations *versus* the number of online accesses (*r* = 0.824) or the Altmetric score (*r* = 0.745), as well as for the number of online accesses *versus* the Altmetric score (*r* = 0.823) (Excel® 2010, Microsoft, RedMond, WA, USA).

Interestingly, among the 100 published articles, the five most quoted articles concerned five different topics that outline the probable future of radiology. Of course, this ranking does not take into account the different exposition time of each article, as recently published papers had a lower probability to be cited. However, the weak inverse correlation of the number of citations with the chronologic order of publication (*r* = -0.228) shows that these articles were indeed the most attractive.

The top ranking is attributed to the article on augmented reality by Philip Pratt et al. [12], from the Department of Surgery and Cancer, Imperial College London, London, and other departments from the same institution, United Kingdom. The article accumulated 17 citations since January 31, 2018. The title well displays the content: *Through the HoloLens™ looking glass: augmented reality for extremity reconstruction surgery using three-dimensional vascular models with perforating vessels*. The authors presented six cases of accurate identification, dissection, and execution of vascular pedunculated flaps during reconstructive surgery of the low extremities using preoperative CT angiography to allow the surgeon to *see through* the patient’s skin and appreciate the underlying anatomy without making a single incision.

The second most quoted article (10 citations since December 22, 2017) regarded the future of CT: the spectral photon-counting era. Daniela Muenzel et al. [13], from the Department of Diagnostic and Interventional Radiology, Klinikum rechts der Isar, Technical University of Munich, and other institutions in Germany and France, reported a proof-of-concept *in silico* study on *Simultaneous dual-contrast multi-phase liver imaging using spectral photon-counting computed tomography*. The authors simulated the complementary distribution in the liver of two contrast agents intravenously injected one after another (a gadolinium- and an iodine-based contrast agent), distinguishing the arterial and portal venous pattern of haemangioma, hepatocellular carcinoma, cyst, and metastasis. Automatic lesion detection performed using a a multidimensional classification algorithm was presented.

The third most quoted paper is a review on the current hot topic of artificial intelligence (AI) in medical imaging [14], coming from my own group at the Department of Radiology, IRCCS Policlinico San Donato, and Università degli Studi di Milano, Italy (9 citations since October 24, 2018). A resident in radiology (Filippo Pesapane), a biomedical engineer (Marina Codari), and myself described the current scenario of research on AI in radiology (driven by the change from traditional *machine learning* to *deep learning*), with MRI and CT as the most involved techniques and neuroradiology as the most involved subspecialty. The main idea of the article is that radiologists, frontrunners of the digital era in medicine, can guide the current era of AI application to healthcare. They will not be replaced by AI because they hold the key of communication of diagnosis, consideration of patient’s values and preferences, medical judgment, quality assurance, education, policy-making, and interventional procedures. The suggestion was to exploit the higher efficiency provided by AI to perform more value-added tasks and to become more visible to patients.

The fourth most quoted paper was authored by Elizabeth J. Sutton et al. [15] from the Department of Radiology, Memorial Sloan Kettering Cancer Center, New York, NY, USA and several other institutions from the same country. It is entitled *Breast MRI radiomics: comparison of computer- and human-extracted imaging phenotypes* and has accumulated 8 citations since November 21, 2017*.* Using the MRI data from The Cancer Genome Atlas project of the National Cancer Institute from 91 breast cancer patients, the authors showed that breast tumour size, shape, and margin extracted by human readers can be replicated by the quantitative computer-extracted radiomics. In the authors’ opinion, as computer algorithms continue to be developed, radiology reports will include quantitative metrics resulting from validated computer algorithms.

Finally, Vito Chianca et al. [16] from the Department of Advanced Biomedical Sciences, Università Federico II, Napoli, and from the Departments of Radiology and Neuroradiology of different university hospitals, Milan, Italy, illustrated the potential of *Diffusion tensor imaging (DTI) in the musculoskeletal and peripheral nerve systems*. The article has had 8 citations since 30 September 2017. After explaining the concept of anisotropy and water diffusion, DTI, and tractography, the authors reviewed the application of DTI to a spectrum of tissues and clinical conditions: normal muscle tissue; muscle contraction and injury; muscular dystrophy; ligaments; peripheral neuropathies; brachial plexus; cubital and carpal tunnel syndromes; sciatic nerve and piriformis syndromes; and nerve tumours. They concluded that DTI and tractography are promising tools providing useful quantitative information about muscular tissue and peripheral nerves as an adjunct to morphological MRI sequences.

In my first editorial in 2017 [17], I discussed the potential role of *European Radiology Experimental* during the “changing times” we live in. Augmented reality, photon-counting CT, AI, radiomics, and advanced MRI are surely part of these changes. Our journal is an open window pointed towards the future.

## Perspectives

One interesting perspective is given by the partially unexpected role that our Methodology section has just begun its growth. It hosted a couple of articles describing rationale, design, and protocol of innovative prospective studies, the first [18] to investigate the integration of positron emission tomography and dual-energy CT systems for staging and image-based radiation therapy planning of lung cancer, and the second [19] to explore the role of standard non-electrocardiographically gated chest CT in cardiac assessment (the Cardiac Pathologies in standard chest CT, CaPaCT, study). This could be a positive perspective for the authors and the journal.

We are now working on a new initiative: *Thematic Series*. These will be original articles and reviews dedicated to hot topics in radiological research, from the lab to the first clinical applications. They will be handled by guest editors. The first one will explore *Myocardial tissue characterization in ischemic heart disease*. Guest editors will be Akos Varga-Szemes and Pal Suranyi from the Medical University of South Carolina, Charleston, USA. It will be launched very soon while other thematic series are already in preparation.

The next stage in our future will require great efforts to get a higher diversity in the worldwide geographic distribution of the origin of the submissions and of the EB members. In addition, we will pay more attention to gender diversity. Only 5 of the 44 EB members are women: 11% of females is a too low rate, whatever reference we take!

## Conclusions

As we have seen, *European Radiology Experimental* had a good commencement. Nothing could have been done without the support of the ESR, which included the key factor of the article processing charge coverage I have mentioned above. But institutions are people who practically help in doing things, advising, supporting, and working as problem-solvers. I want to take the opportunity here to thank from the deep of my heart the fantastic team of the ESR journal office: Stefanie Bolldorf, Irene Christoffel, and Filip Ivkovic. A sincere word of thanks is certainly due to the publisher (SpringerNature), in particular to Isabel Arnold, Christina Kopp, Rhiannon Meaden, and Christopher Ackroyd. Finally, I also want to thank all the EB members, in particular Akos Varga-Szemes, Deputy Editor and a tireless reviewer.

The journal’s motto is “Bringing the future of radiology to you”. This is our mission. Authors, reviewers, and readers are the people who are making this hope a reality.

## Data Availability

All relevant data are reported in the text article and tables.

## Abbreviations

AI: Artificial intelligence
CT: Computed tomography
DTI: Diffusion tensor imaging
EB: Editorial Board
ESR: European Society of Radiology
MRI: Magnetic resonance imaging

## Footnotes

1 Altmetric Support is a source that tracks online conversations around research outputs wherever they happen: books/book chapters, journal articles, presentations, theses/dissertationsr, reports, conference proceedings, reviews, datasets, working papers, grey literature, etc. (https://help.altmetric.com/support/solutions)
